# A Systematic Review of the Role of Robotics in Plastic and Reconstructive Surgery—From Inception to the Future

**DOI:** 10.3389/fsurg.2017.00066

**Published:** 2017-11-15

**Authors:** Thomas D. Dobbs, Olivia Cundy, Harsh Samarendra, Khurram Khan, Iain Stuart Whitaker

**Affiliations:** ^1^Reconstructive Surgery and Regenerative Medicine Research Group (ReconRegen), Institute of Life Science, Swansea University Medical School, Swansea, United Kingdom; ^2^The Welsh Centre for Burns and Plastic Surgery, Morriston Hospital, Swansea, United Kingdom; ^3^Oxford University Medical School, Oxford, United Kingdom; ^4^Department of Plastic Surgery, Birmingham Children’s Hospital, Birmingham, United Kingdom

**Keywords:** robotic surgery, plastic surgery, microsurgery, head and neck, technology, innovation

## Abstract

**Background:**

The use of robots in surgery has become commonplace in many specialties. In this systematic review, we report on the current uses of robotics in plastic and reconstructive surgery and looks to future roles for robotics in this arena.

**Methods:**

A systematic literature search of Medline, EMBASE, and Scopus was performed using appropriate search terms in order to identify all applications of robot-assistance in plastic and reconstructive surgery. All articles were reviewed by two authors and a qualitative synthesis performed of those articles that met the inclusion criteria. The systematic review and results were conducted and reported in accordance with the Preferred Reporting Items for Systematic Reviews and Meta Analysis (PRISMA) guidelines.

**Results:**

A total of 7,904 articles were identified for title and abstract review. Sixty-eight studies met the inclusion criteria. Robotic assistance in plastic and reconstructive surgery is still in its infancy, with areas such as trans-oral robotic surgery and microvascular procedures the dominant areas of interest currently. A number of benefits have been shown over conventional open surgery, such as improved access and greater dexterity; however, these must be balanced against disadvantages such as the lack of haptic feedback and cost implications.

**Conclusion:**

The feasibility of robotic plastic surgery has been demonstrated in several specific indications. As technology, knowledge, and skills in this area improve, these techniques have the potential to contribute positively to patient and provider experience and outcomes.

## Introduction

**Figure 1:**
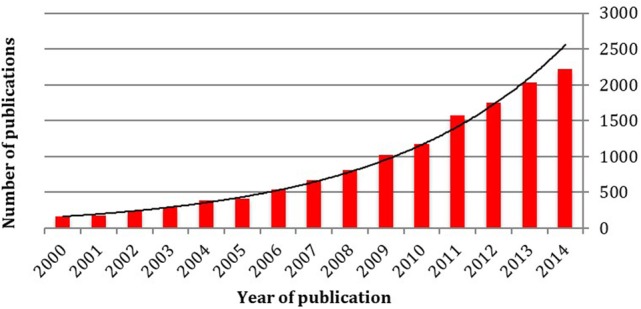
A 15-year literature review of the number of publications relating to robotic surgery demonstrating a highly significant exponential increase. Each column represents the number of papers published in that year, rising from 168 in 2000 to over 2,000 in the year 2014 (Source; Pubmed, searched using the terms “robot” and “surgery” from January 2000 to December 2014).

The use of robotics in surgery has captured the imagination of many. It is a growth area across the breadth of surgical specialties, with many procedures becoming routinely classed as “robot-assisted.” The rapid increase in surgical research involving robotic assistance can be witnessed by the rising number of articles published in consecutive years related to the subject (Figure 1).

Since the first reported use of the daVinci^®^ Surgical Robotic System (Intuitive Surgical, Sunnyvale, CA, USA) in a robotic-assisted laparoscopic cholecystectomy (1), Intuitive Surgical has become the leading force in surgical robotics. The daVinci^®^ robot has been widely implemented in many surgical specialties, from cardiac surgery (2, 3) to gynecology (4, 5). In the USA, 80% of radical prostatectomies are now being performed robotically (6). With updates to the daVinci^®^ robot including a fourth instrument arm, its application is broadening to other specialties such as colorectal surgery (7). The dominance of the daVinci^®^ system is, however, beginning to be challenged with new competitors entering the market.

Plastic and reconstructive surgery is an innovative specialty, often at the forefront of technical innovation within surgery. It is also a specialty that works collaboratively with many other surgical disciplines and, therefore, those practicing it will likely come across advances in robotic surgery in these other specialties. It is, therefore, important for plastic surgeons to embrace this new surgical platform, explore potential uses for it, and learn from those who have already incorporated robotics into their surgical armamentarium.

This systematic review aimed to identify all current reported uses of robotic assistance in plastic and reconstructive surgery, from cadaveric to clinical examples. We have provided and up-to-date list of all areas of interest to the plastic and reconstructive surgeon, evaluating the relevant advantages and disadvantages of the use of robotics in these areas.

## Methods

**Figure 2:**
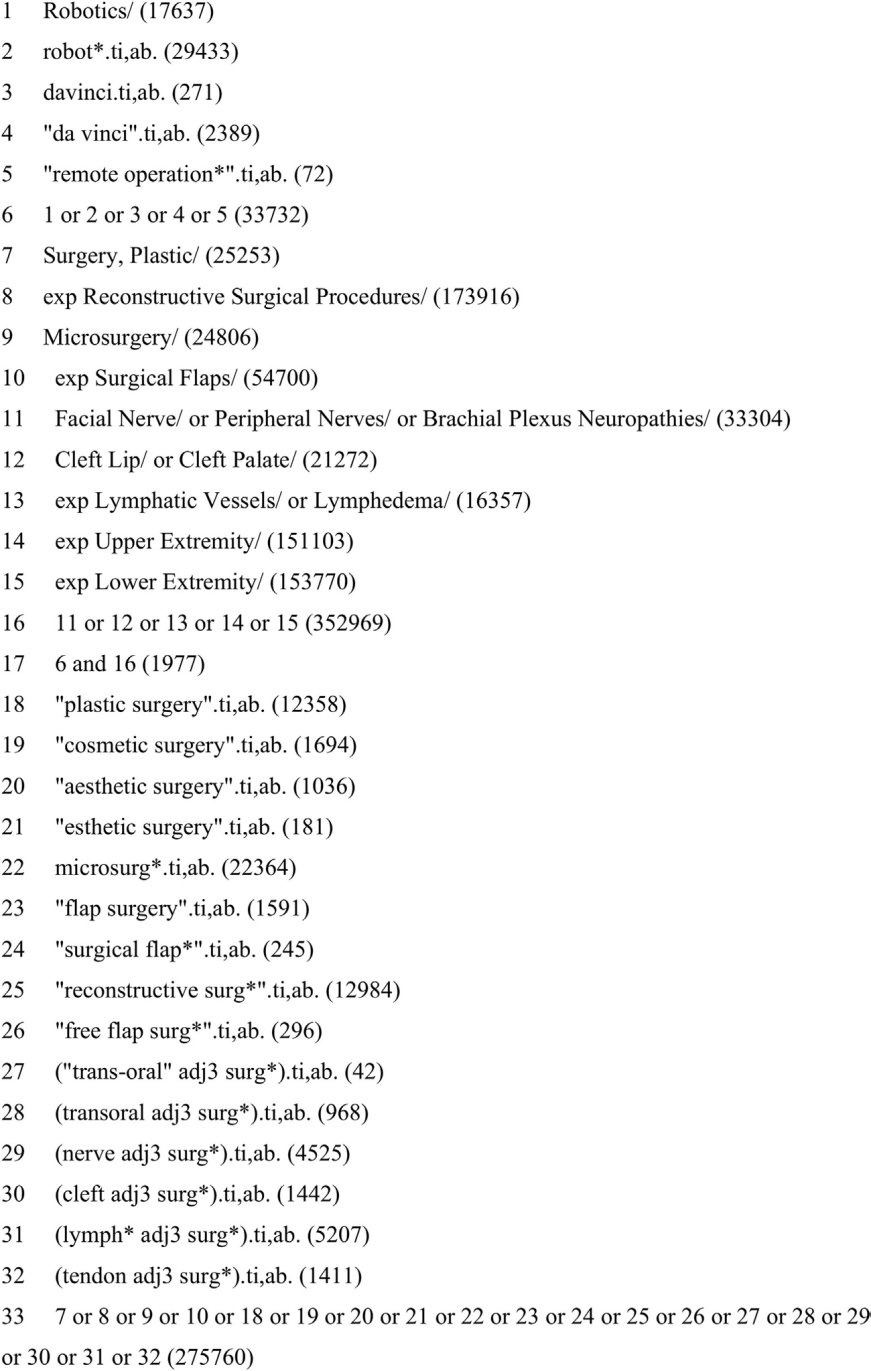
Example search strategy performed in Medline. Searches conducted on 9.5.2017.

A database search was performed to identify all articles describing the use of robotic assistance in plastic and reconstructive surgery. The search strategy was constructed in line with the Preferred Reporting Items for Systematic Reviews and Meta Analysis (PRISMA) guidelines (8) and the Cochrane handbook (9). Key words and Medical Subject Heading terms were combined using Boolean logic and refined with the help of an information specialist (see Figure 2 for an example of the full search strategy). Medline (1946-present), EMBASE (1980-present), and Scopus electronic databases were all searched using the developed search strategy up to May 2017.

All studies identified were downloaded into EndNote V8 for Mac (Clarivate Analytics) and duplicates removed. De-duplicated results were then uploaded to Covidence (www.covidence.org) for screening. Titles and abstracts were reviewed by two independent reviewers (OC and HS) against the inclusion and exclusion criteria and discrepancies resolved through discussion with a third, independent reviewer (TD). Studies were considered eligible for qualitative synthesis if they met the following inclusion criteria:

- the study was published in English
- the study design was one of the following: case reports, case cohorts, case–control and randomized controlled studies. Both prospective and retrospectively designed studies were included.
- the study reports the use of a robotic surgical system for a potential plastic surgery-related operation, with both preclinical and clinical applications included.

Full-text articles of those included studies were subsequently reviewed independently for final inclusion. References were checked for further, un-identified articles, and these were added in if appropriate.

A data extraction sheet was developed to extract the following data from studies: Author, date of publication, location of study, study design, number of operations performed, operations/techniques, outcomes measured. This was piloted on a random sample of papers and subsequently refined. All data were extracted and tabulated using Microsoft Word and Excel (Redmond, Washington, DC, USA).

## Results

**Figure 3:**
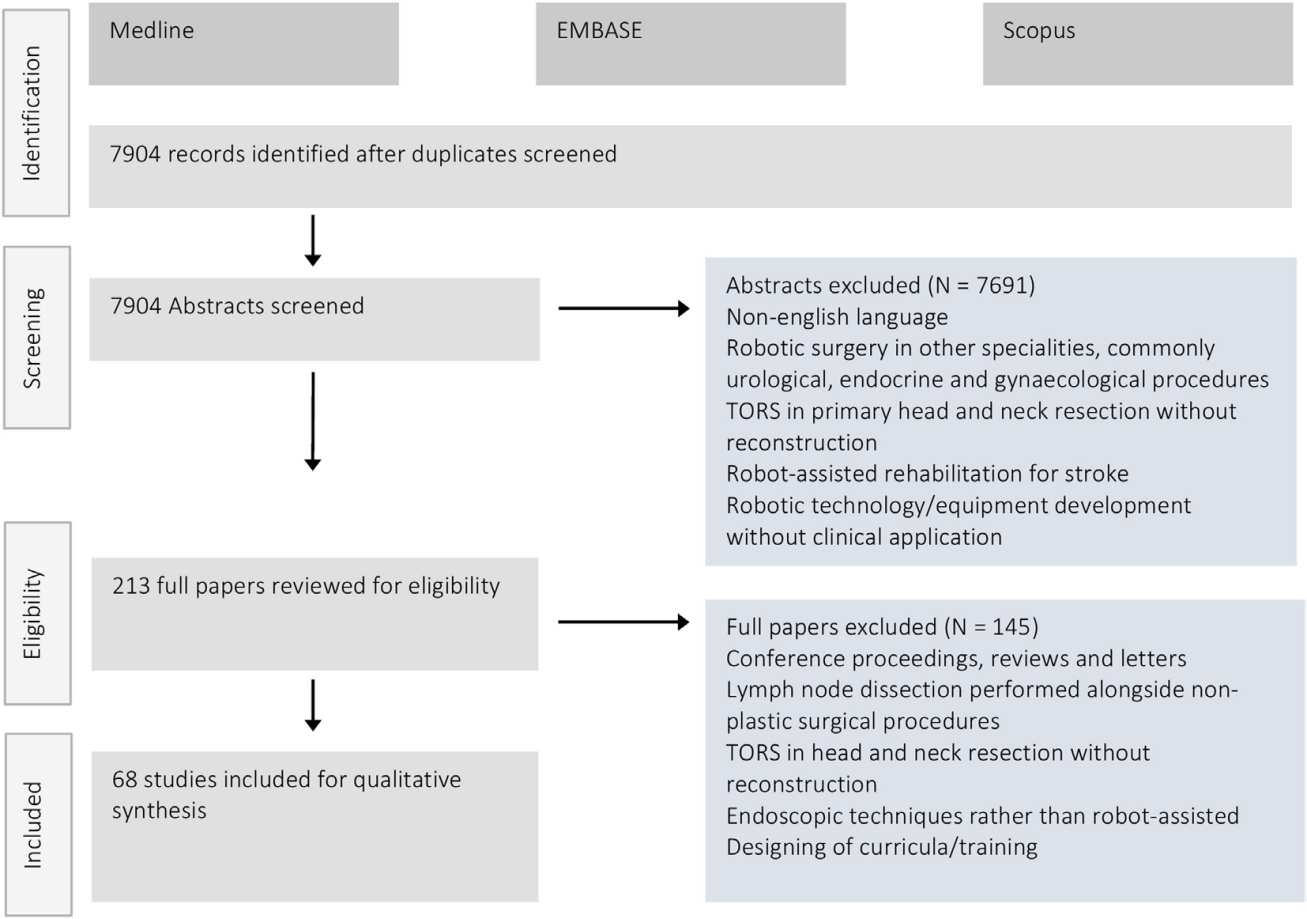
PRISMA flow diagram demonstrating the number of retrieved articles, those screened and final number included in the systematic review after full-text review.

Figure 3 illustrates the PRISMA flow diagram demonstrating the process of article retrieval and screening. A total of 7,904 articles were identified after de-duplication for screening. Of these 213 made it to full-text review. A total of 68 studies met the inclusion criteria and were eligible for inclusion in this systematic review. Included papers were divided into groups based on operative type or body location and a qualitative synthesis of the outcomes reported performed.

### Microsurgery

A total of 13 studies were identified discussing the use of robotics for a microsurgery application (Table 1). Eleven of these were preclinical studies in synthetic, animal, and cadaveric models (10–20) while two were clinical studies (21, 22). Katz et al. performed the first daVinci^®^ system assisted anastomosis in a porcine model in 2005 (10), closely followed by work in canine tarsal and femoral vessels (13). In these studies, they concluded significant advantages such as the elimination of tremor at a microsurgical level, but that the lack of purpose-built microsurgical instruments was an important limitation. Further animal and human cadaveric work cemented the idea that robotically assisted microvascular surgery is both feasible and in some instances potentially beneficial, such as when working at depth and for surgeon comfort (20).

**Table 1:** Preclinical and clinical studies relating to the use of robotics in microvascular procedures.

Reference	Year	Study design	Operations performed	Outcomes reported
**Preclinical studies**				

Katz et al. (10)	2005	Animal model	Arterial and venous anastomoses and free-flap transplantation *(N* = *1 pig)*	All anastomoses grossly patent, confirmed by audible Doppler signals, visibly adequate perfusion of tissues, and arterial bleeding seen after incision distal to the anastomoses 4 h after the procedure

Knight et al. (11)	2005	Animal model	Arterial end-to-end anastomoses (*N* = 31 vs *N* = 30 controls)	A remarkable degree of tremor filtration, but significantly slower operative time. All anastomoses were patent and non-leaking
		Case controlled		

Karamanoukian et al. (12)	2006	Animal tissue samples	Slit arteriotomy and end-to-end arterial anastomoses in procine hearts	The Zeus robotic system is a viable tool for microsurgical vascular reconstruction. It allows for precise movement, lack of hand tremor, enhanced microvascularisation and improved ergonomics, compared to conventional human assistance. The major advantage is the ability of the robot to scale down the surgeon’s movements to a microscopic level

Katz et al. (13)	2006	Animal cadavers	Microvascular anastomoses of tarsal and superficial femoral vessels (*N* = 2 dog cadavers)	All anastomoses were successful and patent postoperatively

Taleb et al. (14)	2008	Animal cadaver	Microvascular anastomoses in rat tail transplantation (*N* = 2)	Immediate and delayed (1 h postoperation) patency of the arterial anastomoses

Ramdhian et al. (15)	2011	Animal tissue samples	Earthworm segment anastomoses (*N* = 15)	The high quality 3D vision allowed by the robotic system was excellent and compensated for loss of tactile feedback. The robotic system eliminated physiological tremor. Motion scaling by the robot improved precision of the surgical gesture

Lee et al. (16)	2012	Live animal models	Femoral artery end-to-end anastomoses (*N* = 20)	Generation of learning curves for robot-assisted microvascular anastomosis. Important aspects of learning identified included starting level, learning plateau and learning rate

Robert et al. (17)	2013	Human cadaver	Radial/ulnar artery dissection and microvascular anastomoses (*N* = 2 cadavers, 4 anastomoses)	Successful anastomoses
				The assembling and disassembling of the vascular clamp were time consuming
				In both cases (radial and ulnar arteries), the 10/0 needle was bent and a second suture had to be used

Alrasheed et al. (18)	2014	Synthetic vessel models	Microvascular anastomoses (*N* = 50)	Successful validation of microsurgical assessment tool and characterization of learning curve
				Proficiency gained by operators over 5 learning sessions

Selber and Alrasheed (19)	2014	Synthetic models	Microvascular anastomoses (*N* = 5 per surgeon)	Definition of a learning curve in microsurgery and the development of a structured assessment of robotic microsurgical skills

Willems et al. (20)	2016	Synthetic microvessel models	Microvascular anastomoses (*N* = 80, vs 80 control)	Manual surgery was superior to robotically assisted microsurgery in technically easy exposures. In difficult exposures (greater depth and lower sidewall angles), however, robotically assisted microsurgery had a shorter surgery time and a higher comfort rating. Objective Structured Assessment of Technical Skills scores were similar to those assessing traditional microsurgery
		Case controlled		

**Clinical studies**				

Boyd et al. (21)	2006	Case cohort, retrospective	Robotic vessel harvest of internal mammary vessels for use in free-flap breast reconstructive procedures (11 muscle-sparing transverse rectus abdominis musculocutaneous (TRAM) flaps, six superior gluteal artery (SGA) flaps, four superficial inferior epigastric artery flaps, and one superior gluteal arterial perforator flap) (*N* = 22 free-flaps, in 20 patients)	Pedicle was harvested with robot-assisted technique
				Microvascular anastomosis via standard technique
				An average pedicle length of 6.7 cm is long enough to allow anastomosis without vein graft

Van der Hulst et al. (22)	2006	Case report	Breast reconstruction with muscle-sparing free TRAM-flap, using robotic arterial anastomosis (*N* = 1)	The time to perform this anastomosis was about 40 min and significantly longer than the standard technique (around 15 min)

*The number of procedures carried out in each study is documented and represented as N number*.

Two clinical examples were identified, with one cohort study by Boyd et al. including 22-patients where the robot was used for harvesting the internal mammary vessels in free breast reconstruction (21). Van der Hulst et al. used the robot to perform the anastomosis, commenting on the increased time taken for this over traditional methods, as would be expected early on in the learning curve (22). As in preclinical studies, the benefits of using the daVinci^®^ robot for performing the microvascular anastomosis include elimination of tremor and motion scaling.

### Muscle Flap Harvest

Traditionally muscle free-flaps are raised through a large incision overlying the muscle belly and are, therefore, a perfect example of where the robot can have marked benefit as minimally invasive harvesting can significantly reduce the size of externally visible scarring. Laparoscopic harvesting has been attempted, but with poor uptake due to difficulties with visualization of the operative field and the inherent limitations of laparoscopic instruments (23, 24). Three human cadaveric studies (25–27) and five clinical reports (28–32) were identified describing the use of the robot for muscle flap harvest (Table 2). In those clinical studies, it is clear that the robot improves visualization, reduces the scar burden and resulted in reduced postoperative pain and hospital stay.

**Table 2:** Preclinical and clinical studies relating to the use of robotics in muscle flap harvest.

Reference	Year	Study design	Operations performed	Outcomes reported
**Preclinical studies**				

Selber (25)	2011	Human cadaver	Latissimus dorsi muscle harvest (*N* = 10 in 8 cadavers)	Successful harvest of all muscles

Patel and Pedersen (26)	2012	Human cadaver	Rectus abdominis muscle dissection and harvest (*N* = 2)	No postoperative complications or surgical-site morbidity

Selber et al. (27)	2012	Human cadaver	Latissimus dorsi muscle harvest and transfer (*N* = 8)	Successful harvest and transfer of all flaps that left no visible incisions, with no major complications
**Clinical studies**				

Patel et al. (28)	2012	Case report	Pedicled myocutaneous latissimus dorsi flap for shoulder reconstruction after sarcoma resection (*N* = 1)	No objective outcomes reported-flap successfully raised robotically One of the limitations is the time/learning curve

Lazzaro et al. (29)	2013	Case report	Intercostal muscle flap after lobectomy (done in conjunction with VATS) (*N* = 1)	Success of surgery—no conversion to open procedures and both patients returned home 5 days postop

Ibrahim et al. (30)	2014	Case series	Rectus abdominus muscle flap harvest (*N* not reported)	Less tissue violation, compared to open technique, resulting in reduced postoperative pain, shorter duration of hospital stay, and more rapid functional recovery

Chung et al. (31)	2015	Case series	Transaxillary gasless robot-assisted latissimus dorsi muscle harvest (3 delayed reconstructions, 4 immediate after nipple sparing mastectomy, 5 corrections of deformity in Poland syndrome) (*N* = 12)	Operating time, general satisfaction, cosmetic satisfaction, scar, and symmetry satisfaction were all outcomes measured *via* survey given to all patients with follow-up longer than months
				Robotic time decreases with experience

Singh et al. (32)	2015	Case series and retrospective review	Extralevator abdominoperineal excision with robotic rectus abdominis flap harvest, for reconstruction after resection of distal rectal adenocarcinoma (*N* = 3)	An incisionless robotic flap harvest with preservation of the anterior rectus sheath obviates the risk of ventral hernia while providing robust tissue closure of the radiated abdominoperineal excision wound

*The number of procedures carried out in each study is documented and represented as N number*.

The traditional approach to rectus muscle harvest is with a large abdominal skin incision. Not only is this cosmetically unappealing but also, in combination with division of the anterior abdominal wall fascia, can result in incisional hernia formation. As robot-assisted colorectal surgery becomes increasingly routine, with the advantages of minimal scarring, reduced conversion to open procedure, reduced time to intestinal motility, and reduced postoperative sexual dysfunction reported (33), it would seem a retrograde step to then introduce a large abdominal wound when harvesting the rectus abdominis muscle for perineal reconstruction. In a case series by Singh et al. the robot was used in tandem with a robotically performed abdominoperineal resection for adenocarcinoma to raise the rectus abdominis flap for reconstruction (32). This produced satisfactory closure of the defect without the risk of a ventral hernia. In these combined procedures the risks associated with entering the abdominal cavity are already present from the colorectal resection and, therefore, one of the major disadvantages of robotically assisted rectus abdominis muscle harvest is not a risk purely implicated through the use of this novel muscle harvest technique.

### Nerve Surgery

A total of eight preclinical studies and five clinical studies were identified, with the majority investigating the role of robotics in brachial plexus work (Table 3) (34–46). Epineural nerve repair using robotic assistance has been shown to be technically feasible in animal models, with the benefits of reduced physiological tremor and improved vision of the surgical field noted (35). Nerve harvest has also been demonstrated to be feasible in cadaveric and animal models (35, 37).

**Table 3:** Preclinical and clinical studies relating to the use of robotics in nerve surgery.

Reference	Year	Study design	Operations performed	Outcomes reported
**Preclinical studies**				

Latif et al. (34)	2008	Animal model	Intercostal nerve grafting for reversal of thoracic sympathectomy (*N* = 1)	Successful anastomosis with no apparent complications

Nectoux et al. (35)	2009	Animal and human tissue samples	Extrafascicular neurolysis, donor nerve dissection and subsequent repair of peripheral nerve (*N* not reported)	The robot removed physiological tremor There was some technical difficulty with the choice and manipulation of the three-dimensional stereoscopic vision enabled a better view and safe and accurate repair of peripheral nerve lesions

Mantovani et al. (36)	2011	Human cadaver	Supraclavicular brachial plexus exploration and nerve graft anastomosis and reconstruction (*N* = 2)	The robot allowed microsurgery to be performed in a very small space with telemanipulation and minimally invasive techniques

Garcia et al. (37)	2012	Human cadaver	Sural nerve graft and neurotisation using the accessory nerve (*N* = 3)	The goals of the operation were achieved without conversion to open surgery. There were no complications

de Melo et al. (38)	2013	Human cadaver	Microsurgical nerve transfer of the branches of the axillary nerve onto the nerve of the long head of the triceps brachii (*N* = 1)	Dissection and transfer achieved successfully

Facca et al. (39)	2014	Human cadaver	Sural nerge graft between C5 root or spinal nerve, and the musculocutaneous nerve (*N* = 8)	Endoscopic treatment of supraclavicular nerve palsy is feasible, however, both sural nerve grafts and C5-6 avulsions were converted to open

Porto de Melo et al. (40)	2014	Animal model	Phrenic nerve harvest and application in brachial plexus surgery (*N* = 1)	Successful nerve harvest

Miyamoto et al. (41)	2016	Animal model	Intercostal nerve harvest for brachial plexus reconstruction (*N* = 3)	Physiological tremor was eliminated and there were no major complications

**Clinical studies**				

Latif et al. (42)	2011	Case study	Intercostal nerve graft harvesting and grafting into sympathetic chain using tension free nerve anastomoses (*N* = 1)	Successful operation, patient discharged one day postoperatively and no sign of Horner’s syndrome on short term follow-up

Coveliers et al. (43)	2013	Case cohort, retrospective	Selective postganglionic thoracic sympathectomy for patients with palmar or axillary hyperhidrosis (*N* = 110 operations in 55 patients)	Of the 55 patients, 53 (96%) had sustained relief of their hyperhidrosis at a median follow-up of 24 months (range, 3 to 36 months), and compensatory sweating was seen in four patients (7.2%)

Naito et al. (44)	2012	Case cohort	The Oberlin procedure of nerve transfer for restoration of elbow flexion (*N* = 4)	At 12 months’ mean follow-up, all patients had recovered to useful elbow flexion, with no sensory/motor deficit in the ulnar nerve territory

Berner (45) (book chapter)	2013	Case series	Repair of brachial plexus injury (*N* = 12)	Considering the microsurgical gesture, all nerve repairs were achieved under excellent conditions Need to convert to open surgery in nine cases

Tigan et al. (46)	2014	Case cohort	Nerve grafting after excision of benign peripheral nerve tumors (*N* = 7)	In postoperative surveys, neuropathic pain halved from 6/10 to 3/10 postop, with no worsening of sensory deficits

*The number of procedures carried out in each study is documented and represented as N number*.

In those clinical studies identified, robotic assistance was successfully used to repair a brachial plexus (45), repair the sympathetic chain to treat Horner’s syndrome (42), perform a thoracic sympathectomy for palmar hyperhidrosis (43), repair a peripheral nerve following tumor excision (46), and undertake an Oberlin procedure (44).

### Upper Limb

Table 4 illustrates those articles relating to procedures in the upper limb, with three preclinical (47–49) and one clinical study identified (50). As with a number of other areas of the body the use of the robot has so far only been for proof of concept and there has yet to be any concrete studies demonstrating a benefit.

**Table 4:** Preclinical and clinical studies relating to the use of robotics in upper limb procedures.

Reference	Year	Study design	Operations performed	Outcomes reported
**Preclinical studies**				

Taleb et al. (47)	2009	Animal cadaver	Humeral cross-section, amputation, and replantation of the left forelimb. Stages done with surgical robot were soft tissue repair and vessel patency tests during limb replantation (not any microvascular procedures) (*N* = 1)	Patency tests were all positive. Venous bleeding demonstrated vascular success of replantation
				The robot removed physiological tremor and allowed for a smaller operating field

Huart et al. (48)	2012	Human cadaver	Kite flap hand surgery (*N* = 1)	Operating time was longer with the robot, but kite flap transfer was successful

Maire et al. (49)	2012	Human cadaver	Removal of left hallux medial hemipulp (with sensory nerve, collateral artery and dorsal vein) and transfer to left thumb radial hemipulp (*N* = 1)	Successful free hallux hemipulp transfer, however, operating time was increased by non-microsurgical moments which could be improved by instrumentation improvement

**Clinical studies**				

Facca and Liverneaux (50)	2010	Case report	Robotic anastomosis of vein grafts for hypothenar hammer syndrome (*N* = 1)	No postoperative problems of note
				Successful cure of vasomotor disorder

*The number of procedures carried out in each study is documented and represented as N number*.

### Trans-Oral Robotic Surgery (TORS)

Trans-oral robotic surgery has allowed head and neck surgeons to treat benign and malignant conditions of the oral cavity and oropharynx avoiding more traditional jaw and lip split approaches, facilitated by the improved access and visualization afforded by the robotic instruments (51, 52). If there is no communication between the oral cavity or oropharynx and neck dissection then the defect could be left to heal by secondary intention; however, in more complex or advanced stages of disease, reconstruction using local flaps or free tissue transfer is required (53). If a jaw split has not been performed, access for satisfactory reconstruction can be almost impossible and thus developing reconstructive techniques using the robot in order to capitalize on the minimized morbidity associated with a TORS resection is of paramount importance.

Trans-oral robotic surgery has become the biggest area for robotic-assisted plastic surgery procedures, with 2 preclinical (54, 55) and 21 clinical studies identified (56–76) (Table 5). Local reconstructive options include the use of the Facial Artery Musculomucosal flap, commonly used in reconstruction of the floor of the mouth and soft palate. Bonawitz and Duvvuri have described using the robot for raising and in-setting the flap with good results (64, 65). Others demonstrated that the use of the robot to perform a musculomucosal advancement flap pharyngoplasty gives good results, both in terms of orocutaneous fistula risk and functional outcomes (60, 61).

**Table 5:** Preclinical and clinical studies relating to the use of robotics in trans-oral robotic surgery (TORS) for a plastic surgery application.

Reference	Year	Study design	Operations performed	Outcomes reported
**Preclinical studies**				

Selber et al. (54)	2010	Coffee cup models, pig cadavers, human cadavers	TORS free radial forearm flap reconstruction of oropharyngeal defect (*N* = 2)	Successful reconstruction of the oropharynx by trans-oral robotic flap inset and microvascular anastomosis
			Robotic microvascular anastomosis	

Smartt et al. (55)	2013	Human cadaver	Superiorly based posterior pharyngeal flap transfer (*N* = 3)	Successful transfer of posterior pharyngeal flaps, with mean surgical time of 113 min. Technically, the learning curve for using the robot telemanipulator was steep
				There was no damage to adjacent structures

**Clinical studies**				

Desai et al. (56)	2008	Case cohort, retrospective analysis	Mucosal flap and pyriform sinus flap reconstructions (*N* = 7)	No intra- or postoperative complications, one patient required tracheostomy

Mukhija et al. (57)	2009	Case series	Radial forearm fasciocutaneous free-flap harvest and reconstruction of oral cavity (*N* = 2)	Successful positioning of the flap, shorter operating time compared to conventional techniques, shorter hospital stay compared to mandibulotomy approach

Selber (58)	2010	Case series	Free-flap reconstruction of oropharynx (radial forearm free-flap, anterolateral thigh flap, facial artery, myomucosal flap), primary closure after tumor resection, and microvascular anastomosis (*N* = 5)	Better access and improved precision within the oropharynx, compared to conventional tecnhiques
				Successful microvascular anastomosis

Garfein et al. (59)	2011	Case report	Radial forearm flap for reconstruction of the tounge base, vallecula and pre-epiglotic space, due to soft tissue and hyoid radionecrosis (*N* = 1)	The patient passed a swallow evaluation after 1 week, and started an oral diet 8 days after the operation
				There was good function showed by video oesophagram 6 week postoperatively

Genden et al. (60)	2011	Prospective non-randomized case–control study	Free-flap reconstruction of oropharynx—sternocleidomastoid free-flap, mucosal mulscular flaps and pharyngoplasty (*N* = 30)	Equivalent rates of loco-regional and distant control of malignancy and better short-term eating ability, compared to conventional techniques
				No major long term sequelae

Genden et al. (61)	2011	Prospective non-randomized case–control study	Musculomucosal advancement flap pharyngoplasty (*N* = 30)	Postoperatively, patients regained excellent function, with near-normal scores on the Functional Oral Intake Scale and Performance Status Scale for Head and Neck Cancer Patients at 1 year after surgery
			Radial forearm free-flap reconstruction	

Bonawitz and Duvvuri (62)	2012	Case cohort, retrospective	Free-flap oropharyngeal reconstruction, with microvascular anastomoses in the tongue base and soft palate (*N* not reported)	No major complications and no flap loss

Longfield et al. (63)	2012	Case series	Robotic reconstruction after resection squamous cell carcinoma of the oropharynx using local and distant free-flaps, with microvascular anastomoses (*N* not reported)	Patients can be safely reconstructed (locally or with free tissue transfer) robotically after TORS

Bonawitz and Duvvuri (64)	2013	Case series	Local random transposition flaps from buccal mucosa, the hard palate or the pharyngeal wall (*N* not reported)	No major complications
			Facial artery musculomucosal (FAMM) flap for larger defects of the soft palate	

Bonawitz and Duvvuri (65)	2013	Case cohort, retrospective	FAMM flap reconstruction after removal of malignant tumors of the soft palate (*N* = 5)	No major complications, no flap loss

Duvvuri et al. (66)	2013	Case cohort, retrospective	Oropharyngeal reconstruction with FAMM free-flaps, ALT free-flaps, radial forearm flaps and uvular flaps (*N* = 12)	No major complications, some minor flap dehiscence, two revision procedures needed (one fistula, one bulky flap)

Hans et al. (67)	2013	Case series	Radial forearm free-flap reconstruction after resection of hypopharyngeal carcinoma (*N* = 2)	A complication of a neck hematoma requiring draining under general anesthesia, no fistulae

Park et al. (68)	2013	Case series, prospective study	Radial forearm muscle free-flap reconstruction of oropharynx (*N* = 7)	No surgery-related complications of infections, viable and functioning free-flaps in all patients, one hundred percent of patients happy with postoperative appearance and could tolerate an oral diet

Song et al. (69)	2013	Case series	Robotic ablation surgery, free-flap reconstruction (radial forearm free-flaps, anterolateral thigh flap), and microvascular anastomosis (*N* = 5)	Flap insetting and microanastomoses were achieved using a specially manufactured robotic instrument
				No complications

De Almeida et al. (70)	2014	Case cohort, retrospective	Velopharyngoplasty reconstructinos with local flaps alone, regional and free-flaps, and secondary healing (*N* = 92)	Good swallowing outcomes, no carotid artery ruptures

Byeon et al. (71)	2015	Case series	Reconstruction and lymph node dissection for head and neck malignancy (*N* = 37)	Good cosmetic outcomes and no major complications

Perrenot et al. (72)	2014	Case series	Infra-hyoid myocutaneous flap reconstructions (*N* = 8)	Good esthetic results
				One case required re-operating due to hemostasis
				No other complications
				Seven out of eight patients tolerated oral feeding postoperatively

Lai et al. (73)	2015	Case cohort	Free radial forearm fasciocutaneous flap reconstruction after resection of oropharyngeal cancer (*N* = 5)	All reconstructive surgeries were successful, with no flap failure or take-backs, no wound infections and no fistulae

Meccariello et al. (74)	2016	Case report	Resection and reconstruction, with temporalis muscle flap, of squamous cell carcinoma of the lateral oropharyngeal wall extending into the soft palate (*N* = 1)	Restoration of a competent velopharyngeal sphincter, with water-tight seal between pharynx and neck
				Timely healing and enhanced postoperative functional results

Gorphe et al. (75)	2017	Non-randomized phase II muti-center prospective trial	FAMM and free ALT flap reconstructions of the oropharynx (*N* = 9)	Robotic surgery proved feasible, and further technological progress in developing robotic systems specifically for trans-oral surgery will be of benefit to patients

Biron et al. (76)	2017	Case–control series	Radial forearm free-flap reconstruction after excision of oropharyngeal squamous cell carcinoma (*N* = 18)	Significantly shorter admission duration and fewer postoperative complications

*The number of procedures carried out in each study is documented and represented as N number*.

In larger or more complex composite defects there is often the requirement for free-flap reconstruction, with specific indications including exposure of the carotid artery, large base-of-tongue defects and defects of the soft palate and tonsillar fossa which cannot be closed with local flap options. The commonest reported free-flap used following TORS resection is the radial forearm flap; however, others such as the anterolateral thigh flap are also described. In the majority of cases the robot was used for flap inset, with authors reporting good access and visualization that allowed a water-tight inset to be achieved and no flap complications despite the lack of a traditional jaw spilled. The robot was also used in a number of studies to perform the vascular anastomosis (58, 62, 63, 69).

### Trans-Oral Robotic Cleft Surgery (TORCS)

Trans-oral robotic cleft surgery is still in its infancy with only three articles identified (77–79) (Table 6); however, it builds upon the same benefit profile achieved by TORS that has been outlined previously for access to the oral cavity and oropharynx in cleft lip and palate patients.

**Table 6:** Preclinical and clinical studies relating to the use of robotics in trans-oral robotic cleft surgery.

Reference	Year	Study design	Operations performed	Outcomes reported
**Preclinical studies**				

Khan et al. (77)	2016	Airway manikin and human cadaver	The Hynes pharyngoplasty (*N* = 1)	With each variation, a subjective assessment (rated as poor, fair, good or excellent) was made for vision and access to either the posterior pharynx or palate, and it was validated by two of the authors for each set-up

Podolsky et al. (78)	2017	Cleft palate simulator test bed	The von Langenbeck cleft palate repair procedure (*N* = 1)	Excellent close up visualization of the anatomy, the ability to articulate the wrist intra-orally (not possible with standard instruments), tremor reduction, better ambidexterity and more precise dissection and tissue manipulation, compared to conventional open techniques

**Clinical studies**				

Nadjmi (79)	2015	Controlled cohort study	The robot was used to dissection and repair the palatine muscles in 10 patients with a cleft of the palate (*N* = 10, 30 controls)	Increased dexterity and operative view using the robot
				Overall operative time was longer using the robot compared to the control group in which the traditional method was used

*The number of procedures carried out in each study is documented and represented as N number*.

### Other Indications

Table 7 demonstrates four other studies identified in the systematic review, which do not fit into the categories above (80–83). Of these indications, it is likely that only lymph node based procedures are likely to progress in the future, with some benefits such as the ability to perform supermicro-surgery an obvious advantage in lymph node transfer.

**Table 7:** Preclinical and clinical studies relating to the use of robotics in other, miscellaneous areas of plastic and reconstructive surgery.

Reference	Year	Study design	Operations performed	Outcomes reported
**Microvascular surgery**				

Dombre et al. (80)	2003	Live animal model	Skin graft (*N* not reported)	Robotically harvested skin samples were of the same quality as manually harvested ones

Taghizadeh et al. (81)	2014	Human cadaver	“Necklift” platysmaplasty—a short incision facelift with concomitant robot-assisted neck lift (*N* = 6)	Successful necklift procedures, with certain areas for improvement in surgical methodology suggested when using robotic systems (hard to interpret)

Shi et al. (82)	2017	Live animal model	Mandibular bone drilling osteotomy (*N* = 1)	The robotically assisted drilling demonstrated more accurate drill positioning, increased stability and accuracy, and relieved surgeon fatigue so as to reduce facial trauma

**Clinical studies**				

Ciudad et al. (83)	2016	Case-report	Tight gastroepiploic lymph node flap (RGE-LNF) for the treatment of lymphedema of the extremities (*N* = 1)	Successful flap harvest, but no postoperative surgical outcomes reported
			Microvascular procedures performed with standard technique	

*The number of procedures carried out in each study is documented and represented as N number*.

## Discussion

In the 30 years since the first robot was used in a surgical procedure the arena of robotic surgery has changed at a breathtaking pace, with the use of the daVinci^®^ robot now common place in some specialties. This revolution has taken longer to impact on the plastic surgery community. It is, therefore, somewhat ironic that it was a plastic surgeon who was at the forefront of robotic and tele-surgery at its inception (84). However, this systematic review has shown that significant developments have been made in the last few years.

The benefits of robotic surgery have been well documented, albeit with no large scale studies, and include reduced blood loss, reduced postoperative pain, faster recovery, and improved cosmesis (85). In relation to plastic and reconstructive surgery the elimination of tremor, greater degree of freedom of the instrument and motion scaling all have the potential to improve the accuracy and reproducibility of microsurgery. The evidence suggests that while the initial learning curve is steep, proficiency in microsurgical skills using the robot can be gained in a short number of sessions (18).

Of the areas identified in this systematic review there are some that are further down the development road than others and some, where the advantages of robotic assistance are greater. For example, with the recent uptake of free-perforator flaps in the field of reconstructive surgery we are beginning to approach the limits of human dexterity at which point the robot may prove to be advantageous. However, to fully exploit this there needs to be focused development in the field of robotic instrument design, expanding the portfolio of micro-instruments. It is our opinion that the potential for robotic head and neck reconstruction is huge and is one of the areas that will most definitely see growth due to the obvious benefits it offers. This will be especially true as the indications for TORS resection continue to widen, resulting in larger and more complex defects. The current limitation to more widespread utilization is instrument design in order to perform microvascular anastomoses and easier inset and it is this area that research should focus. This may also be the case with TORCS. As with cancer resection, there are many circumstances where adequate access to the pharynx and palate in the pediatric cleft patient can pose a significant challenge. The space in which to operate, as well as access for instrumentation, can be severely restricted, especially in cases with abnormal anatomy, poor jaw opening, or anomalies of the nasopharyngeal space. Adequate illumination and visualization can also be difficult. Early work has shown that performing posterior pharyngeal wall surgery using the daVinci^®^ robot is feasible, with benefits such as an improved view, easier dissection, reduced secondary surgical insult and preferential ergonomics for the operating surgeon (77). Its use may also open up avenues of new surgical interventions to areas of the oropharynx that were previously inaccessible.

There is currently less convincing evidence for the use of robotics in areas such as nerve and upper limb surgery. In brachial plexus reconstruction nerve harvest is often required and, therefore, reduced donor site morbidity through robotic harvest, such as with trans-thoracic harvest of intercostal and phrenic nerves, is an area that has the potential for future advancement. It will be important, however, to also demonstrate its safety and cost-effectiveness in order to justify the marginal reductions in scarring when compared to more traditional harvest sites. To date all of the preclinical and clinical studies investigating robotic nerve surgery have demonstrated that it is technically feasible. However, it is still mostly at a proof of concept level and while does have benefits in terms of reduced tremor, it is most likely to be of benefit in difficult to assess areas or when the robot is already being used to perform other parts of the procedure. Finally, at present the indications for the use of robotics in hand surgery are probably more limited than other areas discussed, especially as access is not normally a problem in hand surgery. However, the benefits as discussed for microsurgical anastomosis may prove to be useful in specific indications such as traumatic replantation or congenital reconstruction.

Robotic surgery’s main disadvantage remains the high cost of purchasing and maintaining the equipment. This will undoubtedly improve with time as a greater number of procedures are performed using the robot and the unit cost per operation reduces. A recent comparison of the cost of TORS compared to radiotherapy demonstrated that TORS is currently more expensive; however, this is likely to reduce through the creation of high-volume centers performing TORS (86). It has also been shown that in a center where the learning curve had already been overcome, robotic surgery was cheaper than equivalent open surgery for the surgical treatment of endometrial cancer (87).

Lack of haptic feedback is also often cited as another disadvantage of robotic surgery, with studies demonstrating that operators of augmented robotic surgical systems prefer those with haptic feedback (88). However, other studies such as by Hagen and colleagues who looked at 52 individuals and their perception of haptic feedback while performing robotic surgery demonstrated that visual cues are able to give the perception of haptic feedback, even when true haptic feedback is not present (89). Despite this evidence there is still a tremendous amount of working looking at ways to incorporate haptic feedback into robotic systems, summarized in a review by Okamura (90).

Finally, robotic surgery often results in longer operative times, although this improves with proficiency and in some cases is now comparable to traditional techniques.

The future of robotics in plastic surgery is clearly exciting. Over the last 5 years the range of procedures using the daVinci^®^ robot being attempted by the plastic surgery community has increased significantly and, as technology continues to improve, this will gain further momentum. Of the 68 studies included in this review, only three used a robotic system other than the daVinci^®^. This dominance is beginning to be challenged and while equipment additions such as a micro-forcep is currently available for the daVinci^®^ robot and external companies have developed micro-doppler probes and hydrojet dissectors (91) it will be the development of further microsurgical instruments that will allow greater use of the robot in the field of plastic and reconstructive surgery. The combination of motion scaling and tremor-free instrument manipulation with new instrument design will also allow new avenues in microsurgery that have to date been too technically demanding to be explored. Furthermore, the introduction of a new single port addition to the daVinci^®^ system will allow greater access in trans-oral surgery, improving instrument maneuverability within the tight confines of the intra-oral cavity.

## Conclusion

The potential value of robotic plastic surgery has already been investigated in several specific indications. It is still early days for the field and only time will tell if the use of robotics in plastic surgery is truly of benefit. As the technology, knowledge, and skills in this area improve, it is likely that in specific indications the use of robotic surgery will further contribute positively to patient and provider experience and outcomes. It is, therefore, imperative that the plastic surgery community embraces this new technology platform, but in doing so conducts well-designed, patient-focused research to ensure that it is only being used when there is true benefit to our patients.

## Author Contributions

TD, KK, and IW developed the idea for this paper. TD, OC, and HS developed the search strategy and performed the systematic review and data extraction. TD, KK, IW, OC, and HS wrote the manuscript and all authors edited and agreed the final version.

## Conflict of Interest Statement

The authors declare that the research was conducted in the absence of any commercial or financial relationships that could be construed as a potential conflict of interest.
